# Association of anticoagulant use with clinical outcomes from crizotinib in *ALK* *‐* and *ROS1*‐rearranged advanced non‐small cell lung cancers: A retrospective analysis of PROFILE 1001

**DOI:** 10.1002/cam4.4789

**Published:** 2022-05-05

**Authors:** Terry L. Ng, David C. C. Tsui, Sherry Wang, Tiziana Usari, Tejas Patil, Keith Wilner, David R. Camidge

**Affiliations:** ^1^ Division of Medical Oncology, Department of Medicine University of Ottawa Ottawa Canada; ^2^ Division of Medical Oncology, Department of Medicine University of Colorado School of Medicine Aurora Colorado USA; ^3^ Pfizer Oncology San Francisco California USA; ^4^ Pfizer Milan Italy; ^5^ Pfizer Oncology La Jolla California USA

**Keywords:** *ALK*, crizotinib, lung cancer, *ROS1*, thromboembolism

## Abstract

**Background:**

*ROS1‐* and *ALK*‐rearranged advanced NSCLCs are associated with increased thromboembolic risk. We hypothesized that a prothrombotic phenotype offers an evolutionary advantage to subsets of these cancers. The impact of this phenotype could alter outcomes from targeted therapy.

**Methods:**

In a retrospective analysis of *ROS1‐* and *ALK*‐rearranged NSCLCs treated with crizotinib in a phase 1 trial, we compared progression‐free survival (PFS) and objective response rate (ORR) based on the history of anticoagulation use (a possible surrogate of thromboembolism) at baseline (within 90 days before study enrollment) or within 90 days of study treatment.

**Results:**

Twelve out of 53 (22.6%) *ROS1‐* and 39 out of 153 (25.5%) *ALK*‐rearranged NSCLCs received anticoagulation before or during the trial. Most *ROS1* and *ALK* patients on anticoagulation received low‐molecular‐weight heparin (75% and 64.1%, respectively). In the *ROS1*‐rearranged group, the median PFS (95% CI) values were 5.1 (4.4–14.4) and 29.0 (16.5–48.8) months, and the ORR values were 41.7% (95% CI: 15.2 to 72.3) and 80.5% (95% CI: 65.1 to 91.2) among those with and without anticoagulation treatment, respectively. In the *ALK*‐rearranged group, the median PFS (95% CI) was 7.1 (5.4–7.7) and 12.0 (9.4–18.3) months, and the ORR was 41% (95% CI: 25.6 to 57.9) and 74.3% (95% CI: 65.3 to 82.1) among those with and without anticoagulation, respectively.

**Conclusions:**

Anticoagulation (as a potential surrogate of a prothrombotic subset) in *ROS1‐* and *ALK*‐rearranged NSCLCs may be associated with a lower PFS and ORR to crizotinib.

ClinicalTrial.gov: NCT00585195.

## Graphical abstract

A retrospective analysis of PROFILE 1001, a phase 1 study of 53 ROS1‐ and 153 ALK‐rearranged non‐small cell lung cancer (NSCLC) patients showed that patients started on anticoagulation (as a possible surrogate for thrombosis) had a worse objective response rate and progression‐free survival with crizotinib than those not on anticoagulation.

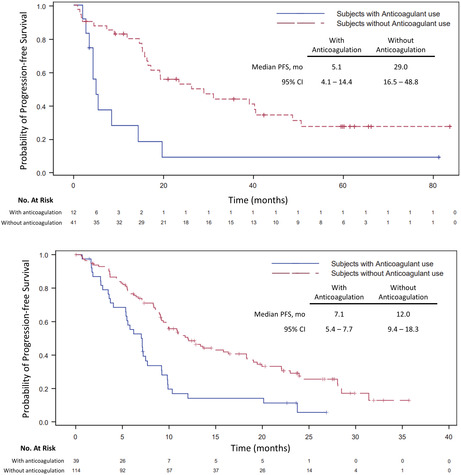

## 1. INTRODUCTION

Although any active cancer has long been associated with an increased risk of thrombosis, multiple studies have reported that the risk of thrombosis in advanced non‐small cell lung cancer (NSCLC) is likely influenced by the specific driver oncogene present in cancer. One study previously reported that patients with *ROS1*‐rearranged NSCLC have significantly elevated rates of arterial or venous thromboses within plus or minus 90 days of the diagnosis of advanced disease compared to *EGFR‐* or *KRAS*‐mutant cases (34.7% versus 13.7% and 18.4%, respectively).^1^ Most of the events occurred within the 90‐day peridiagnostic window, with only a few thromboembolic events captured outside this window. While the rate of thromboembolic events with *ROS1*‐rearranged NSCLC was also numerically higher than with *ALK*‐rearranged NSCLC (22.3%), it was not significantly higher (*p* = 0.229). Similar findings have also been reported in other *ROS1* and *ALK* lung cancer cohorts, further supporting the notion that *ROS1‐*rearranged and *ALK‐*rearranged NSCLCs are specifically associated with an elevated risk of thromboembolic events.^2^, ^3^, ^4^, ^5^, ^6^, ^7^ The elevated rates of thrombosis seen among NSCLC with *ROS1* and *ALK* fusions raise the possibility of a direct oncogene‐related mechanism driving the prothrombotic risk. There is precedence for oncogenes being associated with elevated thrombotic risk. Specifically, *JAK2 V617F* in myeloproliferative disorders^8^, ^9^ and *PML‐RARA* fusions in acute promyelocytic leukemia (APL)^10^, ^11^ are well‐described genetic alterations associated with marked thrombotic risk.

These novel observations associating different driver oncogenes with different clot risk raise multiple additional questions for exploration. Firstly, the mechanistic basis of how different driver oncogenes influence the rate of clotting likely occurs through complex interactions between cancer cells, the endothelial system, and the coagulation cascade, but these remain unclear.^12^, ^13^, ^14^, ^15^, ^16^, ^17^, ^18^, ^19^ Secondly, we hypothesize that these prothrombotic effects in certain oncogene subtypes are not an incidental consequence of cancer, but rather, they represent a selection advantage that cancer cells exploit for growth and survival. This implies that the presence of a thromboembolic event could be associated with different anti‐cancer outcomes including overall survival. Thirdly, if the above hypothesis is true, it also raises the question of whether this prothrombotic phenotype and treatment with anticoagulation could affect more proximal outcomes from targeted therapy such as the objective response rate or progression‐free survival.

In order to investigate these hypotheses further, we explored the clinical outcomes of the *ALK*‐ and *ROS1*‐rearranged NSCLC populations treated with crizotinib (the first licensed *ALK* and *ROS1* inhibitor) within the industry‐sponsored phase I trial, PROFILE 1001, according to the presence or absence of anticoagulant use as a surrogate for a thromboembolic phenotype.

## 2. METHODS

PROFILE 1001 is an open‐label, multicenter, phase 1 trial of crizotinib evaluating the safety and efficacy in an expanded cohort of patients with lung cancers who have *ALK* and *ROS1* rearrangements. Crizotinib 250 mg twice daily was established as the recommended phase 2 dose.

Detailed study eligibility criteria for the original trial have been published previously.^20^, ^21^ Key study exclusion criteria which may have impacted the inclusion of those with elevated thromboembolic risk included a history of central nervous system metastases, significant cardiovascular disease, or cerebrovascular accident including transient ischemic attack within 12 months or pulmonary embolus within 6 months before starting study treatment.

Analyses of the *ROS1* and *ALK* cohorts were conducted separately according to whether patients had or had not received anticoagulation treatment at any time within 90 days before study enrollment or within 90 days of starting study treatment. Progression‐free survival (PFS) and objective response rate on crizotinib were determined in each subgroup. Age, sex, race, weight, height, ECOG performance status, smoking history, and anticoagulation treatment history (timing of anticoagulation and type of anticoagulant drug) in each subgroup were reported descriptively using appropriate summary statistics. All patients who received at least one dose of crizotinib were included in the analyses of PFS. Response‐evaluable patients were defined as all treated patients who had an adequate baseline disease assessment and a minimum of one post‐baseline disease assessment at least 6 weeks from the first dose or who withdrew from the study or had disease progression or death at any time during the study. Overall survival was not available for assessment in the dataset.

Confidence intervals (CIs) for the ORR were estimated using the exact binomial method based on the F‐distribution. Time‐to‐event data were analyzed using the Kaplan–Meier method to estimate median event times, with two‐sided 95% CIs generated using the Brookmeyer–Crowley method. All analyses were performed with SAS statistical software, v9.2 or later (SAS Institute, Inc.).

## 3. RESULTS

Within PROFILE 1001, 53 patients with advanced *ROS1*‐rearranged NSCLC and 153 patients with advanced *ALK*‐rearranged NSCLC were treated. Of these, 12 and 39 patients, respectively, received anticoagulant therapy prior to or during the trial.

The demographics of *ROS1‐* and *ALK*‐rearranged NSCLC patients in the study based on anticoagulant use are reported in Table 1. There were no apparent imbalances in clinical or demographic features based on anticoagulant use in both the *ROS1‐* and *ALK*‐rearranged subgroups.

**TABLE 1:** Demographics of patients with *ROS1‐* and *ALK*‐rearranged NSCLCs based on anticoagulant use

	*ROS1+* (*n* = 53)		*ALK*+ (*n* = 153)	
	With anticoagulant (*n* = 12)	Without anticoagulant (*n* = 41)	With anticoagulant (*n* = 39)	Without anticoagulant (*n* = 114)
	*N* (%)	*N* (%)	*N* (%)	*N* (%)
Age, median (range)	53.0 (35–66)	55.0 (25–81)	54.0 (22–79)	51.0 (25–86)
Age category (years)				
<65	10 (83.3)	28 (68.3)	31 (79.5)	100 (87.7)
≥65	2 (16.7)	13 (31.7)	8 (20.5)	14 (12.3)
Sex (%)				
Male	8 (66.7)	15 (36.6)	20 (51.3)	54 (47.4)
Female	4 (33.3)	26 (63.4)	19 (48.7)	60 (52.6)
Race				
White	7 (58.3)	23 (56.1)	29 (74.4)	68 (59.6)
Black	0	2 (4.9)	2 (5.1)	3 (2.6)
Asian	5 (41.7)	16 (39.0)	5 (12.8)	38 (33.3)
Japanese	0	0	0	15 (13.2)
Korean	2 (16.7)	11 (26.8)	3 (7.7)	20 (17.5)
Chinese	2 (16.7)	2 (4.9)	0	2 (1.8)
Other	1 (8.3)	3 (7.3)	2 (5.1)	1 (0.9)
Other	0	0	3 (7.7)	5 (4.4)
Mean weight, kg (SD)	80.2 (19.03)	69.4 (14.32)	73.7 (17.58)	69.4 (14.97)
Mean height, kg (SD)	174.5 (8.54)	165.1 (9.55)	167.0 (9.12)	168.4 (10.43)
Smoking history				
Never smoked	10 (83.3)	30 (73.2)	26 (66.7)	83 (72.8)
Ex‐smoker	2 (16.7)	11 (26.8)	12 (30.8)	31 (27.2)
Smoker	0	0	1 (2.6)	0
ECOG status				
0	3 (25.0)	20 (48.8)	12 (30.8)	45 (39.5)
1	9 (75.0)	20 (48.8)	22 (56.4)	55 (48.2)
2	0	1 (2.4)	5 (12.8)	13 (11.4)
3	0	0	0	1 (0.9)

In the *ROS1*‐rearranged group, anticoagulation treatments included low‐molecular‐weight heparin (LMWH), heparin, warfarin, fondaparinux, or a direct oral anticoagulant (DOAC) (Table S1). Of the 12 patients on anticoagulation, nine patients were already on anticoagulation at baseline, two patients started anticoagulation during study treatment, and one discontinued all anticoagulation before starting crizotinib (i.e., they were on anticoagulation at screening but had discontinued by the start of crizotinib therapy).

In the *ALK*‐rearranged group, anticoagulation treatments included LMWH, heparin, warfarin, and fondaparinux (Table S1). Of the 39 patients on anticoagulation, 25 patients were already on anticoagulation at baseline, 12 patients started anticoagulation during study treatment, and two discontinued all anticoagulants before starting crizotinib.

This was a retrospective analysis on available datasets. The data cutoff date for patients with ROS1‐rearranged NSCLC was June 30, 2018 and for patients with *ALK*‐rearranged NSCLC was April 13, 2012, with some patients still on treatment.

**FIGURE 1:**
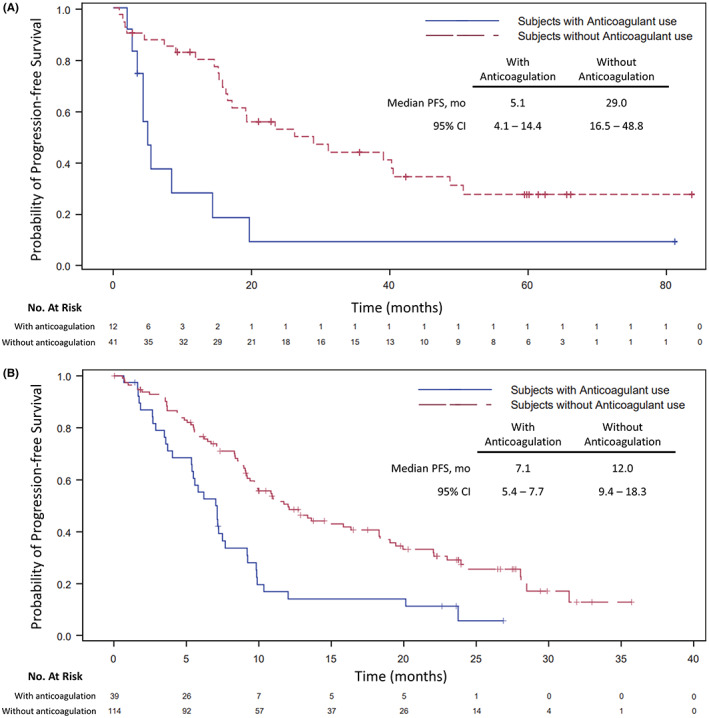
Progression‐free survival in *ROS1*‐rearranged NSCLC based on anticoagulant use. Progression‐free survival in *ALK*‐rearranged NSCLC based on anticoagulant use

In the *ROS1*‐rearranged group, among those without anticoagulation, 26 (63.4%) of 41 subjects had experienced a PFS event, two of which were recorded as death without objective progression (Table S2). Among those with anticoagulation, 10 (83.3%) of 12 subjects had experienced a PFS event, one of which was recorded as death without objective progression. The median PFS (95% CI) among those with and without anticoagulation was 5.1 (4.4–14.4) and 29 (16.5–48.8) months, respectively (Figure 1).

In the *ALK*‐rearranged group, among those without anticoagulation, 77 (67.5%) of 114 subjects had experienced a PFS event, five of which were recorded as death without objective progression (Table S2). Among those with anticoagulation, 34 (87.2%) of 39 subjects had experienced a PFS event, 10 of which were recorded as death without objective progression. The median PFS (95% CI) among those with and without anticoagulation was 7.1 (5.4–7.7) and 12 (9.4–18.3) months, respectively (Figure 1).

The objective response rate in the *ROS1*‐rearranged group among those with and without anticoagulation was 41.7% (95% CI: 15.2–72.3), with 5 out of 12 (41.7%) achieving a partial response, and 80.5% (95% CI: 65.1–91.2), with 6 out of 41 (14.6%) achieving a complete response and 27/41 (65.8%) achieving a partial response, respectively (Table 2).

**TABLE 2:** Best objective response for patients with *ROS1‐* and *ALK*‐rearranged NSLCCs based on anticoagulant use

	*ROS1+* (*n* = 53)		*ALK*+ (*n* = 152^a^)	
	With anticoagulant (*n* = 12)	Without anticoagulant (*n* = 41)	With anticoagulant (*n* = 39)	Without anticoagulant (*n* = 113)
	*N* (%)	*N* (%)	*N* (%)	*N* (%)
Best Overall response				
Complete response	0 (0)	6 (14.6)	1 (2.6)	4 (3.5)
Partial response	5 (41.7)	27 (65.9)	15 (38.5)	80 (70.8)
Stable disease	6 (50.0)	4 (9.8)	17 (43.6)	21 (18.6)
Objective progression	1 (8.3)	2 (4.9)	3 (7.7)	4 (3.5)
Early death^b^	0 (0)	1 (2.4)	1 (2.6)	3 (2.7)
Indeterminate	0 (0)	1 (2.4)	2 (5.1)	1 (0.9)
Objective response rate (CR + PR)	5 (41.7)	33 (80.5)	16 (41.0)	84 (74.3)
95% confidence interval	(15.2–72.3)	(65.1–91.2)	(25.6–57.9)	(65.3–82.1)

^a^ One patient out of the total *n* = 153 was not evaluable.

^b^ Early death is death within 42 days (6 weeks) from the first dose

The objective response rate in the *ALK*‐rearranged group among those with and without anticoagulation was 41% (95% CI: 25.6–57.9), with 1 out of 39 (2.6%) achieving a complete response and 15 out of 39 (38.5%) achieving a partial response, and 74.3% (95% CI: 65.3–82.1), with 4 out of 113 (3.5%) achieving a complete response and 80 out of 113 (70.8%) achieving a partial response, respectively (Table 2).

## DISCUSSION

The interaction between cancer and the hemostatic system is bidirectional. Tumors promote a procoagulant state, but components of the hemostatic system may also play a role in tumor growth, angiogenesis, invasion, and metastases.^22^ Evidence supporting this hypothesis comes from the interplay between protease‐activated receptors (PARs) and the coagulation system. PAR‐1 is expressed on cancer cells and activated by thrombin.^23^Activated PAR‐1 appears to play a crucial role in prometastatic activities through β1‐integrin and matrix metalloprotease^24^, ^25^ and proangiogenic programs.^23^, ^26^, ^27^ Consequently, thrombin generation (through TF‐VIIa) could create a positive feedback loop for cancer growth, invasion, and vascularization. With the recognition of the differing risk of thromboembolic events among oncogenic driver subgroups in advanced NSCLC, the concept that a prothrombotic phenotype may directly impact cancer outcomes, assuming this phenotype reflects some evolutionarily advantageous strategies of cancer, must also be considered. Therefore, we sought to determine whether there is an association between therapeutic anticoagulation (as a surrogate of thrombosis) and clinical outcomes within the initial trial of crizotinib in advanced *ROS1‐* and *ALK*‐rearranged NSCLCs.

Considering the rate of anticoagulation as a surrogate for the rate of thromboembolic events, 22.6% with *ROS1‐* and 25.5% with *ALK*‐rearranged NSCLCs in this study had documented anticoagulant use within 90 days before or after the study enrollment. In the *ALK*‐rearranged NSCLC cohort, the percentage of anticoagulation was similar to our previously reported thromboembolic rate (22.3%). Although the percentage of anticoagulation in the *ROS1* cohort was lower than the thromboembolic rate in our previous report (34.7%), it is still consistent with a higher frequency of thromboembolic events compared to other molecular subtypes of lung cancer.^1^ Furthermore, the difference between the *ROS1* cohorts could be explained by a larger margin of error associated with a smaller sample size in the PROFILE 1001 study (*n* = 53).

In this study, PFS outcomes in the subgroup that used anticoagulation were consistently worse relative to the overall trial population and the subgroup without anticoagulation history. In the overall trial population, crizotinib in *ROS1*‐rearranged NSCLC (*n* = 53) led to a median PFS of 19.3 months (95% CI: 15.2–39.1).^21^ In comparison, the *ROS1* subgroups with and without anticoagulation had a median PFS of 5.1 months (95% CI, 4.4–14.4) and 29 months (95% CI: 16.5–48.8), respectively. Similarly, in the previously published *ALK* cohort (*n* = 143), the median PFS was 9.7 months (95% CI: 7.7–12.8).^20^ In our analyses, which included an additional 10 patients, the median PFS (95% CI) in the subgroups with and without anticoagulation use was 7.1 months (5.4–7.7) and 12 months (9.4–18.3), respectively.

Whether the differential PFS outcomes seen in association with anticoagulation reflect an impact on death related to thromboses or other non‐cancer‐related etiologies as opposed to an effect on the progression of cancer per se, has to be considered. The proportion of PFS events attributable to death in the absence of documented radiographic progression was numerically higher among those on anticoagulation than among those not on anticoagulation in the *ALK*‐rearranged group (10/34 [29%] versus 5/77 [6%], respectively), suggesting that a worse PFS might partly be attributed to non‐cancer‐related deaths. However, the same trend was not apparent in the *ROS1*‐rearranged group; the proportion of PFS events attributable to death in the absence of documented radiographic progression was 1 out of 10 (10%) among those on anticoagulation versus 2/26 (8%) among those not on anticoagulation.

Worsened PFS outcomes in the anticoagulation subgroup may also simply reflect thrombosis as a surrogate for a greater disease burden and likelihood for a shorter disease control period as well as worse general health status due to comorbid medical conditions which may not be fully captured by ECOG performance status. However, the tumor response rate to crizotinib in the *ALK*‐rearranged cohort was also lower in the anticoagulation subgroup than in the non‐anticoagulated subgroup, and a similar trend was also observed in the cohort of *ROS1*‐rearranged NSCLC. These observations suggest an association between anticoagulant use (a surrogate of thrombotic events) and worsened targeted therapy outcomes, and support the hypothesis that worsened PFS in the cohort with anticoagulant use reflects fundamentally different tumor biology in patients with a prothrombotic phenotype.

Potential weaknesses of our analyses include the fact that patients with a new pulmonary embolism occurring within 6 months or a cerebrovascular accident within 12 months prior to the start of the study were excluded from the study. These criteria may have excluded some patients with a prothrombotic phenotype. In addition, although the vast majority of *ROS1* and *ALK* patients with a history of anticoagulant use (83.3% and 69.2%) were started on anticoagulation prior to starting crizotinib within the anticoagulation subgroup, patients who commenced anticoagulation within 90 days of starting crizotinib were also included. Despite this, it should be noted that crizotinib has not been significantly associated with increased thromboembolic events despite its routine use for many years. For example, the first‐line PROFILE 1014 phase III trial conducted in *ALK*‐rearranged NSCLC showed that the rate of grade 3 or 4 pulmonary embolism was 8% for the crizotinib arm and 7% for the chemotherapy arm.^28^ Similarly, the PROFILE 1007 phase III trial showed that the rate of grade 3 or 4 pulmonary embolism was 5% for the crizotinib arm and 2% for the chemotherapy arm in the second‐line setting.^29^

Multiple previous trials have evaluated the effect of anticoagulation on response rate and survival outcomes across different tumor types, most of which have suggested, if anything, an association with better outcomes.^30^, ^31^, ^32^ In our cohort of *ALK‐* and *ROS1*‐rearranged NSCLCs, as the use of anticoagulation (predominantly LMWH) was associated with worsened PFS and ORR, this supports the possibility of a unique subset of patients with a prothrombotic phenotype that is resistant to targeted therapy. One hypothesis is that any evolutionary advantage to cancer from this phenotype might manifest upstream of the site of the anticoagulant’s mechanisms of action, and therefore, the associated inferior clinical outcomes could not be reversed with anticoagulation treatment.

Although our study was a retrospective analysis, the data on clinical outcomes were generated from a prospective, multicenter trial with uniform surveillance imaging schedule and formalized tumor response measurements using RECIST 1.0. Regarding other limitations, although the reasons for anticoagulation were not available, LMWH was used in 10 out of 12 cases of anticoagulation in the *ROS1* cohort and in 32 out of 39 cases in the *ALK* cohort. LMWH is more commonly used in the treatment of venous thromboembolic events and less commonly used in non‐thrombotic indications such as stroke prevention for atrial fibrillation. Also, our study may have underestimated the total prevalence of thrombotic events due to missed asymptomatic venous thromboembolic events. The number of patients in each cohort on anticoagulation was relatively small, and the balance of other potential molecular and clinical risk factors between those with and without anticoagulation such as tumor burden or anatomic location of metastases was unknown. Finally, we were unable to generate data on any impact of the use of anticoagulation on overall survival, but this should be a focus in later studies.

Despite these caveats, based on the data generated, we propose that thromboembolic events potentially reflect a previously unidentified subset of *ROS1‐* and *ALK*‐rearranged NSCLC patients with a prothrombotic phenotype that confers a cancer cell survival advantage via a mechanism that is upstream of the targets of commonly used anticoagulants that therefore cannot be overcome by commonly used therapeutic anticoagulation. The observations from our study should be validated in larger studies in patients with a de novo diagnosis of metastatic *ROS1‐* and *ALK‐*rearranged NSCLCs to control for lead time bias and for more comprehensive tumor molecular typing. Future studies should also focus on elucidating the mechanism of how these oncogenes increase the risk of thromboembolic events in some or all patients and whether interfering with these pathways at different points in their initiation may improve treatment outcomes. The association between anticoagulant use and PFS in our study was greatest in the *ROS1* cohort, which is also the subtype of NSCLC with the highest known peridiagnostic clot risk. Our other dataset addressed the association in *ALK*‐rearranged NSCLC, another molecular subtype that has also been associated with a high clot risk as reported in multiple study cohorts. It would also be interesting to ask whether the association of thrombosis/anticoagulation with worse outcome is unique to *ROS1‐* and *ALK*‐rearranged NSCLCs by conducting similar analyses in other molecularly defined subtypes of NSCLC treated with relevant targeted therapies. Finally, our study also raises the prospect that the presence or absence of baseline thrombosis / anticoagulation should be explored as a potential stratification factor in future interventional clinical trials of targeted therapy to improve the prognostic balance between experimental and control arms.

## AUTHOR CONTRIBUTIONS

T.L.N., D.C.C.T., and D.R.C. conceived the study and wrote the first draft of the manuscript. Pfizer (S.W., T.U., and K.W.) provided patient data. All authors analyzed the data, reviewed, and approved the final manuscript.

## CONFLICT OF INTEREST

Dr. Ng reports grants from Takeda Oncology, personal fees from ARIAD, personal fees from Takeda Oncology, and personal fees from Boehringer Ingelheim, outside the submitted work. Dr. Tsui has nothing to disclose. Dr. Wang reports personal fees from Pfizer, outside the submitted work. Dr. Usari reports personal fees from Pfizer, outside the submitted work. Dr. Patil reports personal fees from Roche/Genentech, personal fees from AstraZeneca, personal fees from Guidepoint Global, personal fees from FCB Health, and personal fees from Aptitude Health, outside the submitted work. Dr. Wilner reports personal fees from Pfizer Inc, outside the submitted work. Dr. Camidge reports grants from Takeda Oncology, personal fees from Takeda Oncology, personal fees from Pfizer, and personal fees from Roche, outside the submitted work.

## ETHICAL APPROVAL STATEMENT

The protocol was approved by the research ethics committee at each study site, and all patients provided written informed consent before enrollment.

## Supporting information


Table S1‐S2

## Data Availability

Upon request and subject to review, Pfizer will provide the data that support the findings of this study. Subject to certain criteria, conditions, and exceptions, Pfizer may also provide access to the related individual de‐identified participant data. See https://www.pfizer.com/science/clinical‐trials/trial‐data‐and‐results for more information.

## References

[1] Ng TL , Smith DE , Mushtaq R , et al. ROS1 gene rearrangements are associated with an elevated risk of peridiagnosis thromboembolic events. J Thorac Oncol. 2019;14(4):596‐605. doi:10.1016/j.jtho.2018.12.001 30543838

[2] Zer A , Moskovitz M , Hwang DM , et al. ALK‐rearranged non‐small‐cell lung cancer is associated with a high rate of venous thromboembolism. Clin Lung Cancer. 2017;18(2):156‐161. doi:10.1016/j.cllc.2016.10.007 27913214

[3] Zugazagoitia J , Biosca M , Oliveira J , et al. Incidence, predictors and prognostic significance of thromboembolic disease in patients with advanced ALK‐rearranged non‐small cell lung cancer. Eur Respir J. 2018;51(5):1702431 10.1183/13993003.02431-2017 29563169

[4] Al‐Samkari H , Leiva O , Dagogo‐Jack I , et al. Impact of ALK rearrangement on venous and arterial thrombotic risk in NSCLC. J Thorac Oncol. 2020;15(9):1497‐1506. doi:10.1016/j.jtho.2020.04.033 32437899

[5] Dou F , Zhang Y , Yi J , et al. Association of ALK rearrangement and risk of venous thromboembolism in patients with non‐small cell lung cancer: a prospective cohort study. Thromb Res. 2020;186:36‐41. doi:10.1016/j.thromres.2019.12.009 31864154

[6] Chiari R , Ricciuti B , Landi L , et al. ROS1‐rearranged non‐small‐cell lung cancer is associated with a high rate of venous thromboembolism: analysis from a phase II, prospective, multicenter, two‐arms trial (METROS). Clin Lung Cancer. 2020;21(1):15‐20. doi:10.1016/j.cllc.2019.06.012 31607443

[7] Alexander M , Pavlakis N , John T , et al. A multicenter study of thromboembolic events among patients diagnosed with ROS1‐rearranged non‐small cell lung cancer. Lung Cancer. 2020;142:34‐40. doi:10.1016/j.lungcan.2020.01.017 32087434

[8] Bellucci S , Michiels JJ . The role of JAK2 V617F mutation, spontaneous erythropoiesis and megakaryocytopoiesis, hypersensitive platelets, activated leukocytes, and endothelial cells in the etiology of thrombotic manifestations in polycythemia vera and essential thrombocythemia. Semin Thromb Hemost. 2006;32(4 Pt 2):381‐398. doi:10.1055/s-2006-942759 16810614

[9] Falanga A , Marchetti M , Vignoli A , et al. V617F JAK‐2 mutation in patients with essential thrombocythemia: relation to platelet, granulocyte, and plasma hemostatic and inflammatory molecules. Exp Hematol. 2007;35(5):702‐711. doi:10.1016/j.exphem.2007.01.053 17577920

[10] Breen KA , Grimwade D , Hunt BJ . The pathogenesis and management of the coagulopathy of acute promyelocytic leukaemia. Br J Haematol. 2012;156(1):24‐36. doi:10.1111/j.1365-2141.2011.08922.x 22050876

[11] Rashidi A , Silverberg ML , Conkling PR , Fisher SI . Thrombosis in acute promyelocytic leukemia. Thromb Res. 2013;131(4):281‐289. doi:10.1016/j.thromres.2012.11.024 23266518

[12] Boccaccio C , Comoglio PM . Genetic link between cancer and thrombosis. J Clin Oncol. 2009;27(29):4827‐4833. doi:10.1200/JCO.2009.22.7199 19738115

[13] Rak J , Yu JL , Luyendyk J , Mackman N . Oncogenes, trousseau syndrome, and cancer‐related changes in the coagulome of mice and humans. Cancer Res. 2006;66(22):10643‐10646. doi:10.1158/0008-5472.CAN-06-2350 17108099

[14] Payne H , Ponomaryov T , Watson SP , Brill A . Mice with a deficiency in CLEC‐2 are protected against deep vein thrombosis. Blood. 2017;129(14):2013‐2020. doi:10.1182/blood-2016-09-742999 28104688 PMC5408561

[15] Riedl J , Preusser M , Nazari PM , et al. Podoplanin expression in primary brain tumors induces platelet aggregation and increases risk of venous thromboembolism. Blood. 2017;129(13):1831‐1839. doi:10.1182/blood-2016-06-720714 28073783 PMC5823234

[16] Geddings JE , Hisada Y , Boulaftali Y , et al. Tissue factor‐positive tumor microvesicles activate platelets and enhance thrombosis in mice. J Thromb Haemost. 2016;14(1):153‐166. doi:10.1111/jth.13181 26516108 PMC4715578

[17] Rak J , Milsom C , May L , Klement P , Yu J . Tissue factor in cancer and angiogenesis: the molecular link between genetic tumor progression, tumor neovascularization, and cancer coagulopathy. Semin Thromb Hemost. 2006;32(1):54‐70. doi:10.1055/s-2006-933341 16479463

[18] Zwicker JI , Liebman HA , Neuberg D , et al. Tumor‐derived tissue factor‐bearing microparticles are associated with venous thromboembolic events in malignancy. Clin Cancer Res. 2009;15(22):6830‐6840. doi:10.1158/1078-0432.CCR-09-0371 19861441 PMC2783253

[19] Falanga A , Panova‐Noeva M , Russo L . Procoagulant mechanisms in tumour cells. Best Pract Res Clin Haematol. 2009;22(1):49‐60. doi:10.1016/j.beha.2008.12.009 19285272

[20] Camidge DR , Bang YJ , Kwak EL , et al. Activity and safety of crizotinib in patients with ALK‐positive non‐small‐cell lung cancer: updated results from a phase 1 study. Lancet Oncol. 2012;13(10):1011‐1019. doi:10.1016/S1470-2045(12)70344-3 22954507 PMC3936578

[21] Shaw AT , Riely GJ , Bang YJ , et al. Crizotinib in ROS1‐rearranged advanced non‐small‐cell lung cancer (NSCLC): updated results, including overall survival, from PROFILE 1001. Ann Oncol. 2019;30(7):1121‐1126. doi:10.1093/annonc/mdz131 30980071 PMC6637370

[22] Kuderer NM , Ortel TL , Francis CW . Impact of venous thromboembolism and anticoagulation on cancer and cancer survival. J Clin Oncol. 2009;27(29):4902‐4911. doi:10.1200/JCO.2009.22.4584 19738120 PMC2799059

[23] Snyder KM , Kessler CM . The pivotal role of thrombin in cancer biology and tumorigenesis. Semin Thromb Hemost. 2008;34(8):734‐741. doi:10.1055/s-0029-1145255 19214911

[24] Radjabi AR , Sawada K , Jagadeeswaran S , et al. Thrombin induces tumor invasion through the induction and association of matrix metalloproteinase‐9 and beta1‐integrin on the cell surface. J Biol Chem. 2008;283(5):2822‐2834. doi:10.1074/jbc.M704855200 18048360 PMC2805198

[25] Shi X , Gangadharan B , Brass LF , Ruf W , Mueller BM . Protease‐activated receptors (PAR1 and PAR2) contribute to tumor cell motility and metastasis. Mol Cancer Res. 2004;2(7):395‐402. 15280447

[26] Nierodzik ML , Karpatkin S . Thrombin induces tumor growth, metastasis, and angiogenesis: evidence for a thrombin‐regulated dormant tumor phenotype. Cancer Cell. 2006;10(5):355‐362. doi:10.1016/j.ccr.2006.10.002 17097558

[27] Schaffner F , Ruf W . Tissue factor and protease‐activated receptor signaling in cancer. Semin Thromb Hemost. 2008;34(2):147‐153. doi:10.1055/s-2008-1079254 18645919

[28] Solomon BJ , Kim DW , Wu YL , et al. Final overall survival analysis from a study comparing first‐line Crizotinib versus chemotherapy in ALK‐mutation‐positive non‐small‐cell lung cancer. J Clin Oncol. 2018;36(22):2251‐2258. doi:10.1200/JCO.2017.77.4794 29768118

[29] Shaw AT , Kim DW , Nakagawa K , et al. Crizotinib versus chemotherapy in advanced ALK‐positive lung cancer. N Engl J Med. 2013;368(25):2385‐2394. doi:10.1056/NEJMoa1214886 23724913

[30] Lebeau B , Chastang C , Brechot JM , et al. Subcutaneous heparin treatment increases survival in small cell lung cancer. "Petites Cellules" group. Cancer. 1994;74(1):38‐45. doi:10.1002/1097-0142(19940701)74:1<38::aid-cncr2820740108>3.0.co;2-e 8004580

[31] Altinbas M , Coskun HS , Er O , et al. A randomized clinical trial of combination chemotherapy with and without low‐molecular‐weight heparin in small cell lung cancer. J Thromb Haemost. 2004;2(8):1266‐1271. doi:10.1111/j.1538-7836.2004.00871.x 15304029

[32] Kuderer NM , Khorana AA , Lyman GH , Francis CW . A meta‐analysis and systematic review of the efficacy and safety of anticoagulants as cancer treatment: impact on survival and bleeding complications. Cancer. 2007;110(5):1149‐1161. doi:10.1002/cncr.22892 17634948

