# Supplementary material for: Association of anticoagulant use with clinical outcomes from crizotinib in ALK ‐ and ROS1‐rearranged advanced non‐small cell lung cancers: A retrospective analysis of PROFILE 1001
Source: Cancer Med. 2022 May 5;11(23):4422–9. doi: 10.1002/cam4.4789 (PMC9741966; doi:10.1002/cam4.4789)
Supplement: Supplementary file 1 — Table S1‐S2 [file CAM4-11-4422-s001.docx]

***Supplementary table 1***

Temporal relationship between anticoagulation and crizotinib initiation, and class of anticoagulation therapy used.

|  | *ROS1+* (n=53) | *ALK*+ (n=153) |
| --- | --- | --- |
|  | N (%) | N (%) |
| Subjects with anticoagulant at the time of crizotinib start | 9 (17.0) | 25 (16.3) |
| Subjects who started all anticoagulants after the start of crizotinib | 2 ( 3.8) | 12 (7.8) |
| Subjects who discontinued all anticoagulants before the start of crizotinib | 1 ( 1.9) | 2 (1.3) |
| Class of initial anticoagulants used |  |  |
| Low molecular weight heparin (LMWH) | 9 (17.0) | 25 (16.3) ^b^ |
| Heparin | 0 | 2 (1.3) ^c^ |
| Warfarin | 1 (1.9) | 7 (4.6) ^c^ |
| Fondaparinux | 1 (1.9) ^a^ | 5 (3.3) ^d^ |
| DOAC | 1 (1.9) | 0 |

1. One patient switched to heparin transiently before switching back to fondaparinux
2. One patient switched to heparin transiently before switching back to LMWH
3. One patient switched to LMWH
4. One patient switched to heparin transiently then switched to LMWH

***Supplementary table 2***

Details about events/censored in the PFS analyses, and timed PFS

|  | *ROS1+* (n=53) | | *ALK*+ (n=153) | |
| --- | --- | --- | --- | --- |
|  | With anticoagulant (n=12) | Without anticoagulant (n=41) | With anticoagulant (n=39) | Without anticoagulant (n=114) |
|  | N (%) | N (%) | N (%) | N (%) |
| Number with event | 10 (83.3) | 26 (63.4) | 34 (87.2) | 77 (67.5) |
| Type of event |  |  |  |  |
| Objective progression | 9 (75.0) | 24 (58.5) | 24 (61.5) | 72 (63.2) |
| Death without objective progression | 1 (8.3) | 2 (4.9) | 10 (25.6) | 5 (4.4) |
|  |  |  |  |  |
| Number censored | 2 (16.7) | 15 (36.6) | 5 (12.8) | 37 (32.5) |
| PFS at 6 months (%) (95% CI) | 37.5 (11.7 - 63.8) | 87.7 (73.0 - 94.7) | 55.3 (38.3 - 69.4) | 76.6 (67.6 - 83.4) |
|  |  |  |  |  |
| Median PFS in months (95% CI) | 5.1 (4.4 - 14.4) | 29.0 (16.5 - 488) | 7.1 (5.4 - 7.7) | 12.0 (9.4 - 18.3) |
